# Automated Diabetic Retinopathy Detection Using Horizontal and Vertical Patch Division-Based Pre-Trained DenseNET with Digital Fundus Images

**DOI:** 10.3390/diagnostics12081975

**Published:** 2022-08-15

**Authors:** Sabiha Gungor Kobat, Nursena Baygin, Elif Yusufoglu, Mehmet Baygin, Prabal Datta Barua, Sengul Dogan, Orhan Yaman, Ulku Celiker, Hakan Yildirim, Ru-San Tan, Turker Tuncer, Nazrul Islam, U. Rajendra Acharya

**Affiliations:** 1 Department of Ophthalmology, Firat University Hospital, Firat University, Elazig 23119, Turkey; 2 Department of Computer Engineering, Faculty of Engineering, Kafkas University, Kars 36000, Turkey; 3 Department of Ophthalmology, Elazig Fethi Sekin City Hospital, Elazig 23280, Turkey; 4 Department of Computer Engineering, Faculty of Engineering, Ardahan University, Ardahan 75000, Turkey; 5 School of Management & Enterprise, University of Southern Queensland, Darling Heights, QLD 4350, Australia; 6 Faculty of Engineering and Information Technology, University of Technology Sydney, Ultimo, NSW 2007, Australia; 7 Department of Digital Forensics Engineering, Technology Faculty, Firat University, Elazig 23119, Turkey; 8 Department of Cardiology, National Heart Centre Singapore, Singapore 169609, Singapore or; 9 Duke-NUS Medical Centre, Singapore 169857, Singapore; 10 Glaucoma Faculty, Bangladesh Eye Hospital & Institute, Dhaka 1209, Bangladesh; 11 Ngee Ann Polytechnic, Department of Electronics and Computer Engineering, Singapore 599489, Singapore; 12 Department of Biomedical Engineering, School of Science and Technology, SUSS University, Singapore 599494, Singapore; 13 Department of Biomedical Informatics and Medical Engineering, Asia University, Taichung 41354, Taiwan

**Keywords:** diabetic retinopathy, patch division, deep feature extraction, transfer learning, neighborhood component analysis, support vector machine

## Abstract

Diabetic retinopathy (DR) is a common complication of diabetes that can lead to progressive vision loss. Regular surveillance with fundal photography, early diagnosis, and prompt intervention are paramount to reducing the incidence of DR-induced vision loss. However, manual interpretation of fundal photographs is subject to human error. In this study, a new method based on horizontal and vertical patch division was proposed for the automated classification of DR images on fundal photographs. The novel sides of this study are given as follows. We proposed a new non-fixed-size patch division model to obtain high classification results and collected a new fundus image dataset. Moreover, two datasets are used to test the model: a newly collected three-class (normal, non-proliferative DR, and proliferative DR) dataset comprising 2355 DR images and the established open-access five-class Asia Pacific Tele-Ophthalmology Society (APTOS) 2019 dataset comprising 3662 images. Two analysis scenarios, Case 1 and Case 2, with three (normal, non-proliferative DR, and proliferative DR) and five classes (normal, mild DR, moderate DR, severe DR, and proliferative DR), respectively, were derived from the APTOS 2019 dataset. These datasets and these cases have been used to demonstrate the general classification performance of our proposal. By applying transfer learning, the last fully connected and global average pooling layers of the DenseNet201 architecture were used to extract deep features from input DR images and each of the eight subdivided horizontal and vertical patches. The most discriminative features are then selected using neighborhood component analysis. These were fed as input to a standard shallow cubic support vector machine for classification. Our new DR dataset obtained 94.06% and 91.55% accuracy values for three-class classification with 80:20 hold-out validation and 10-fold cross-validation, respectively. As can be seen from steps of the proposed model, a new patch-based deep-feature engineering model has been proposed. The proposed deep-feature engineering model is a cognitive model, since it uses efficient methods in each phase. Similar excellent results were seen for three-class classification with the Case 1 dataset. In addition, the model attained 87.43% and 84.90% five-class classification accuracy rates using 80:20 hold-out validation and 10-fold cross-validation, respectively, on the Case 2 dataset, which outperformed prior DR classification studies based on the five-class APTOS 2019 dataset. Our model attained about >2% classification results compared to others. These findings demonstrate the accuracy and robustness of the proposed model for classification of DR images.

## 1. Introduction

Diabetes mellitus is a chronic medical condition characterized by high blood sugar levels, which, over time, induce progressive target organ damage in the heart, kidney, brain, nerves, and eyes [1,2]. In diabetic retinopathy (DR), blood vessels in the retina can rupture within the retina, producing small retinal hemorrhages (non-proliferative DR), which can leak, producing retinal edema, which reduces visual acuity, or new vessels can grow in front of the retina (proliferative disease), which can cause major bleeding into the eye [3,4]. DR can be stratified into non-proliferative diabetic retinopathy (NPDR) and proliferative diabetic retinopathy (PDR), the latter being a much more serious accelerated deteriorating phase [5]. Symptoms of DR include blurred vision, difficulty seeing in the dark, progressive and even sudden vision loss [6]. DR affects about 80% of people who have had diabetes for 20 years or more [7] and is the commonest cause of vision loss in individuals aged 20 to 74 years [8]. By 2030, the number of people with DR will reach 191 million [9]. In addition, comorbidities, such as high blood pressure and hypercholesterolemia, common in diabetes, can exacerbate the risk of vision loss in DR [10,11]. Fortunately, vision loss can be averted with early diagnosis and treatment before the disease becomes too advanced [12]. For this reason, it is recommended that patients with diabetes undergo routine annual fundal photography, where retinal images are acquired and then interpreted by ophthalmologists. However, manual interpretation is subject to inter- and intra-observer variability and may be inefficient for high-throughput screening. To address this, automated machine-learning-enabled computer-aided diagnostic systems [13,14] have been introduced to screen DR on fundal photographs (Table 1).

We aimed to develop a fast, automated machine-learning-based DR classification that can be deployed in the clinic to assist doctors in screening for DR efficiently. This will facilitate early diagnosis and treatment of DR and prevent vision loss among diabetic patients. Toward this aim, a new dataset was collected for training the model, which comprised novel patch-based image division, DenseNet201 [32] for feature extraction, neighborhood component analysis (NCA) [33] for feature selection, and cubic support vector machine (SVM) [34] for classification.

The novelties of our research are given as follows. (i) We collected a new fundus im-age dataset to classify PDR, NPDR, and healthy. (ii) A new nonfixed-size patch division method has been proposed to extract local features in the feature extraction phase. (iii) Pretrained deep features have been generated by using our proposed patch division method and a pretrained DenseNet201. NCA chooses the top informative features, and classification results have been obtained by deploying an SVM classifier. In this respect, a new rectangular patch division-based deep-feature engineering model has been presented.

The contributions of our model are:

- A new DR image dataset was collected, on which the developed model was tested.
- Novel division of the image into horizontal and vertical patches enabled downstream multilevel deep-feature extraction.
- Feature extraction was performed at the last fully connected, and global average pooling layers of DenseNet201, a deep network architecture, and the most discriminative features were selected with NCA.
- The model was trained and tested on our new dataset and the established open-access Asia Pacific Tele-Ophthalmology Society (APTOS) dataset [35]. As a result, the model attained excellent 94.06% and 91.55% classification accuracy rates on our new dataset using robust 80:20 hold-out validation and 10-fold cross-validation (CV) strategies, respectively.

## 2. Material and Method

### 2.1. Material

#### 2.1.1. New Diabetic Retinopathy Dataset

**Figure 1:**
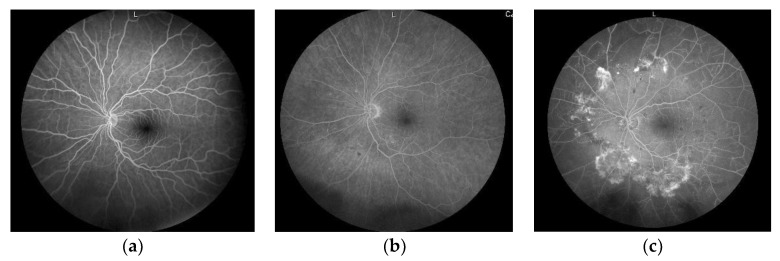
Sample images from the new diabetic retinopathy dataset: (**a**) normal, (**b**) NPDR, (**c**) PDR.

A new dataset was retrieved from the clinical database of digitized fundal photographs of diabetic patients who attended the Ophthalmology Department, the Firat University Hospital, Turkey. The hospital ethics board had approved the retrospective data collection. This dataset comprised a total of 2355 images that had been labeled by ophthalmologists into three classes: 366 normal (15.5%); 1022 NPDR (43.4%); and 967 PDR (41.1%). Sample images of this dataset are given in Figure 1.

#### 2.1.2. APTOS 2019 Diabetic Retinopathy Dataset

**Figure 2:**
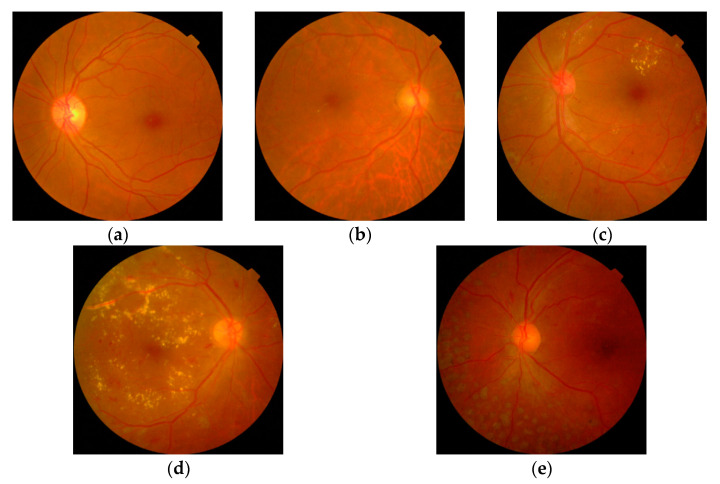
Sample images from APTOS 2019 dataset: (**a**) normal, (**b**) mild DR, (**c**) moderate DR, (**d**) severe DR, (**e**) PDR.

This is an open-access dataset [35] comprising 3662 images that had been labeled into five classes: 1805 normal (49.3%); 370 mild DR (10.1%); 999 moderate DR (27.3%); 193 severe DR (5.3%); and 295 PDR (8.1%). Sample images of these classes are given in Figure 2. To ameliorate the data imbalance, an analysis scenario Case 1 was created by amalgamating mild, moderate, and severe classes into a single NPDR class, i.e., 1805 normal (49.3%); 1562 NPDR (42.7%); and 295 PDR (8.1%), which possessed a structure similar to our new dataset. In the second analysis scenario, Case 2, classification was made using the existing five classes.

### 2.2. Method

**Figure 3:**
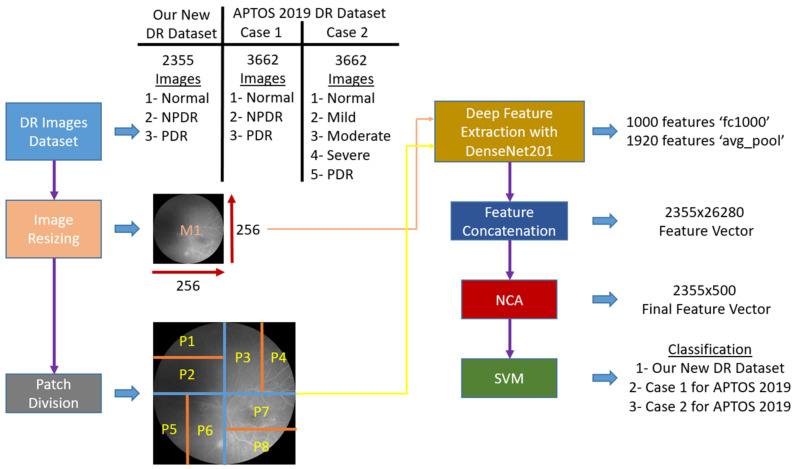
Block diagram of the proposed patch-based model. Each input image is first resized to 256 × 256, then divided into four equal nonoverlapping quadrants. Each quadrant is further subdivided into horizontal or vertical patches. Pretrained DenseNet201 is used to generate 2920 features from the main image (M1), as well as each of the horizontal (P1, P2, P7, and P8) and vertical (P3, P4, P5, and P6) patches, which are all concatenated to form a final feature vector of length 26,280 (= 9 × 2920). Neighborhood component analysis (NCA) is used to select the top 500 features, which are fed to a support vector machine (SVM) for classification. APTOS, Asia Pacific Tele-Ophthalmology Society; DR, diabetic retinopathy; NPDR, non-proliferative diabetic retinopathy; PDR, proliferative diabetic retinopathy.

Inspired by the success of image recognition methods, such as vision transformer [36] and multilevel perceptron mixer [37], in which image patch division allowed multilevel deep-feature extraction, we built a DR image classification model using a novel scheme of horizontal and vertical patch divisions. The developed model applied transfer learning using the pretrained DenseNet201 architecture to extract deep features from both original images, as well as the subdivided patches. This efficient approach generated a high volume of features with compressed training time. Further, a simple yet effective NCA algorithm was used to select the most discriminative features, which were then fed to a standard shallow classifier, SVM. The block diagram of the model is shown in Figure 3, and the pseudocode in Algorithm 1. Details of the various steps are explained in the following sections.
**Algorithm 1.** Pseudocode of the proposed patch-based model**Input:** DR image dataset (drd). **Output:** Classification results (cr)00: Load drd. 01: **for** k = 1 to noi
**do** // Herein, noi is the number of images 02:  $Im=drd_{k};$ // Read each image (Im) from dataset 03:  Resize the image to 256 × 256. 04:  $feat\left(k,1:2920\right)=DenseNet201\left(Im\right);$ // Generate deep features from the main image // using “fc1000” and “avg_pool” layers 05:  Divide DR image into horizontal and vertical patches $\left(P\right)$ // Herein, P is patches 06:  cnt=0 // Counter for patches 07:  **for** i = 1 to 8 **do //** There are 8 patches 08:   $feat\left(k,c\times 2920+1:2920\times \left(c+1\right)\right)=DenseNet201\left(P_{i}\right);$ 09:   c=c+1; 10:  **end for i** 11: **end for k** 12: Normalize feat using min-max normalization 13: Apply NCA to feat and calculate indexes (ind) 14: Select the top 500 features using ind 15: Export selected features to the cubic SVM classifier. 16: Obtain classification results (cr) with 10-fold CV and 80:20 hold-out validation

The key point of Algorithm 1 is Line 6. In this step, the main image is divided into nonoverlapping quadrants, and then subdivided into horizontal and vertical patches so that local features can be later extracted from the latter in the horizontal and vertical fields, respectively. The patch division is carried out in a predetermined fixed scheme: horizontal direction in the upper left and lower right quadrants; vertical direction in the upper right and lower left quadrants.

#### 2.2.1. Feature Extraction

**Figure 4:**
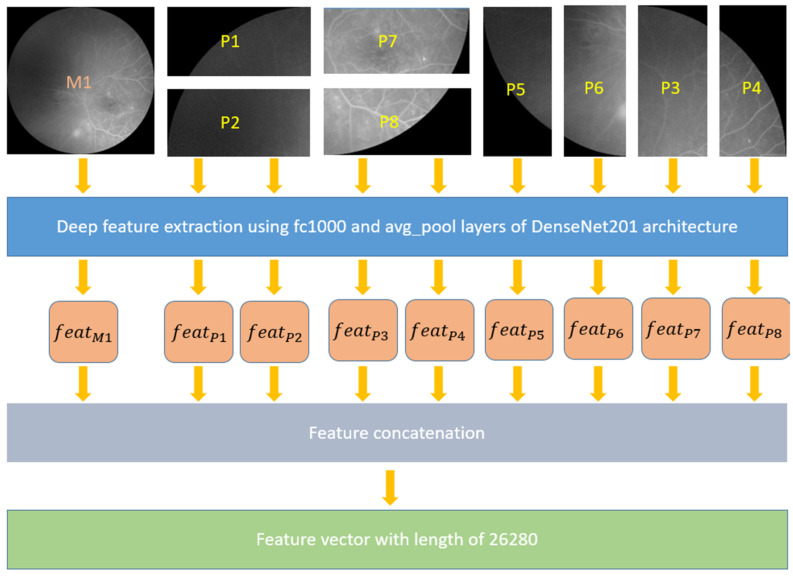
Block diagram of the feature extraction process. The resized main image (MI) is divided into four quadrants. Depending on its relative position in the MI, the quadrants are divided into horizontal (P1, P2, P7, and P8) and vertical (P3, P4, P5, and P6) patches.

First, the fundal photograph images were resized to 256 × 256 (most of the CNNs and patch-based model, such as vision transformers and Swin transformers, have used resizing to obtain fixed-sized images/patches; thus, we used resizing operator), then divided into four equal quadrants (128 × 128). Each quadrant was further subdivided into either horizontal (128 × 64) or vertical (64 × 128) patches depending on its relative position in the original image (Figure 4), i.e., eight patches were generated from a single DR image. Next, feature extraction was performed using the DenseNet201 architecture via a transfer learning approach. DenseNet201 [32] is a 201-layer open-access convolutional neural network that has been pretrained on image classification using the >1,000,000-image ImageNet1k dataset [38]. Specifically, the fully connected and average pooling layers of DenseNet201 extracted 1000 and 1920 features, respectively, from the main DR image and each of the eight patches. All the extracted features were concatenated into one final feature vector of a length of 26,280 (= 9 × (1000 + 1920)) for each input DR image. Herein, 9 defines the numbers of used images (1 raw image + 8 patches), and 1000 and 1920 are defined as the number of generated features using fc1000 and avg_pool layers of the used pretrained DenseNet201, respectively.

Detailed steps of feature extraction are listed below:

Step 1: Divide the image into horizontal and vertical patches. Herein, the size of the patches is 128 × 64 for horizontal and 64 × 128 for vertical patches.

Step 2: Extract features from the main image, and for each patch use “fc1000” and “avg_pool” layers of pretrained DenseNet201.

Step 3: Concatenate the deep features and obtain the final feature vector of length 26,280.

#### 2.2.2. Feature Selection

NCA [33], a well-known effective feature selection function, was used in the feature selection phase of our model. The optimal number of selected features was determined by trial and error. NCA eliminated redundant features for each input image from the large final feature vector of length 26,280 and selected the top 500 most discriminative features with the best weights. Detailed steps of feature selection are listed below:

Step 4: Apply min–max normalization to the feature vector.

Step 5: Apply NCA to the generated feature vector with a length of 26,280 and generate qualified indexes.

Step 6: Choose the most discriminative 500 features from feature vectors based on the qualified indexes.

#### 2.2.3. Classification

SVM [34], a standard shallow classifier, was used to assess the model’s classification performance. A third-degree polynomial kernel (cubic SVM) was chosen, along with 10-fold CV and 80:20 hold-out validation strategies, to validate the model.

Step 7: Classify the selected 500 features by applying to the SVM classifier with a 10-fold CV and 80:20 hold-out technique.

## 3. Results

### 3.1. Experimental Setup

The model was implemented using MATLAB 2021b programing platform on a server with the following specifications: Intel Xeon @2.70 GHz, 256 GB main memory, and 500 GB external memory. The proposed model was tested on our new three-class DR dataset, as well as the three- and five-class datasets of Case 1 and Case 2, respectively, derived from the APTOS 2018 dataset.

Moreover, details of this architecture are given below.

Patch division: eight nonfixed-sized patches have been created.

Feature extraction: the last fully connected and global average pooling layers of the pretrained DenseNet201. In the feature extraction phase, nine (a raw image and nine patches) inputs have been used. Thus, our proposed feature extractor generates 26,280 (=9 × 2920) features.

Feature selection: the most informative 500 features from the generated 26,280 features are selected by deploying NCA.

Classification: the selected/chosen top 500 features have been utilized as input of the SVM classifier to obtain results.

To evaluate the classification performance of the model, confusion matrixes were constructed and standard performance metrics, such as accuracy, precision, recall, F1-score, Cohen’s kappa, and geometric mean, were computed.

### 3.2. Results

**Figure 5:**
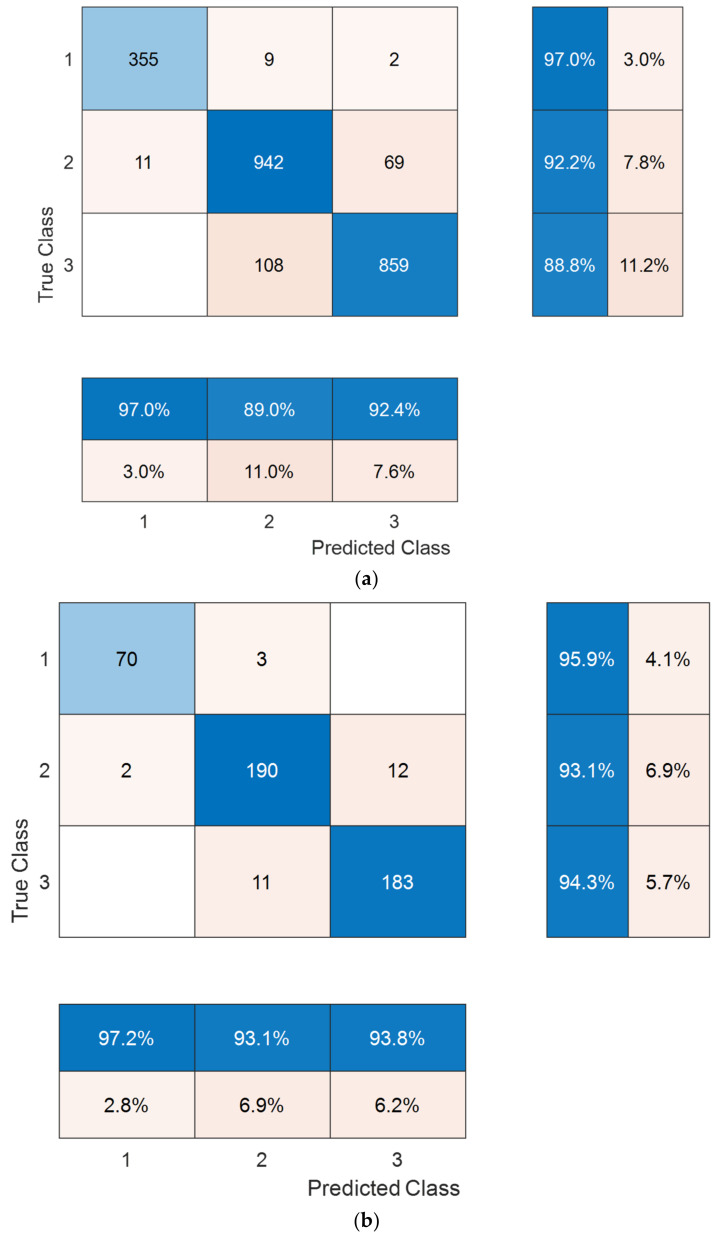
Confusion matrixes for our new dataset obtained using 10-fold cross-validation (**a**) and 80:20 hold-out validation (**b**). Class names: 1, normal; 2, non-proliferative diabetic retinopathy; 3, proliferative diabetic retinopathy.

The results are presented stratified by the DR dataset (new dataset, Case 1, and Case 2) and validation strategy (80:20 hold-out validation versus 10-fold CV). For our new DR dataset, class-wise results for both validation strategies are depicted in confusion matrixes (Figure 5), and performance metrics for overall three-class classification are summarized in Table 2. The best class-wise accuracy was observed in the normal class with either validation strategy. Overall three-class classification performance was numerically superior with hold-out validation across all performance metrics compared with 10-fold CV, e.g., 94.06% versus 91.55% classification accuracy.

**Figure 6:**
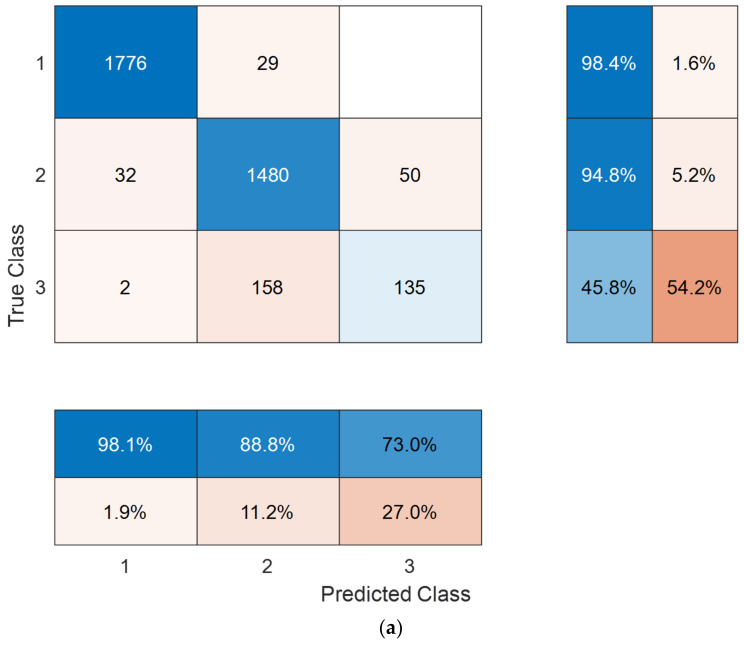

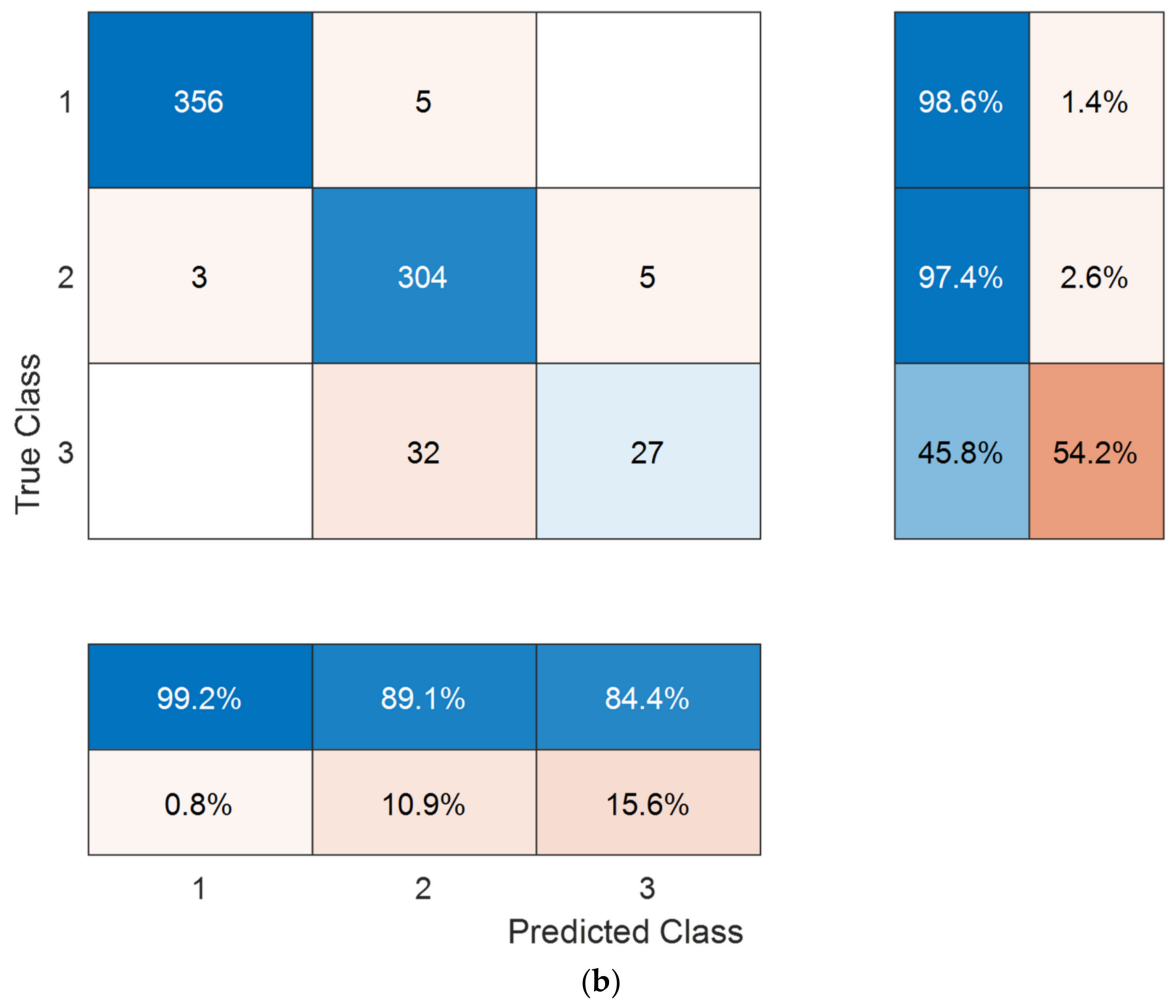
Confusion matrixes for our new dataset obtained using 10-fold cross-validation (**a**) and 80:20 hold-out validation (**b**). Class names: 1, normal; 2, non-proliferative diabetic retinopathy; 3, proliferative diabetic retinopathy.

The salutary results were validated on the external APTOS 2019 dataset. For Case 1, the results were similar to those obtained on our new dataset. The best class-wise accuracy was observed in the normal class with either validation strategy (Figure 6). Again, overall three-class classification performance was numerically superior with hold-out validation across all performance metrics compared with 10-fold CV, e.g., 93.85% versus 92.6% classification accuracy as presented in Table 3.

**Figure 7:**
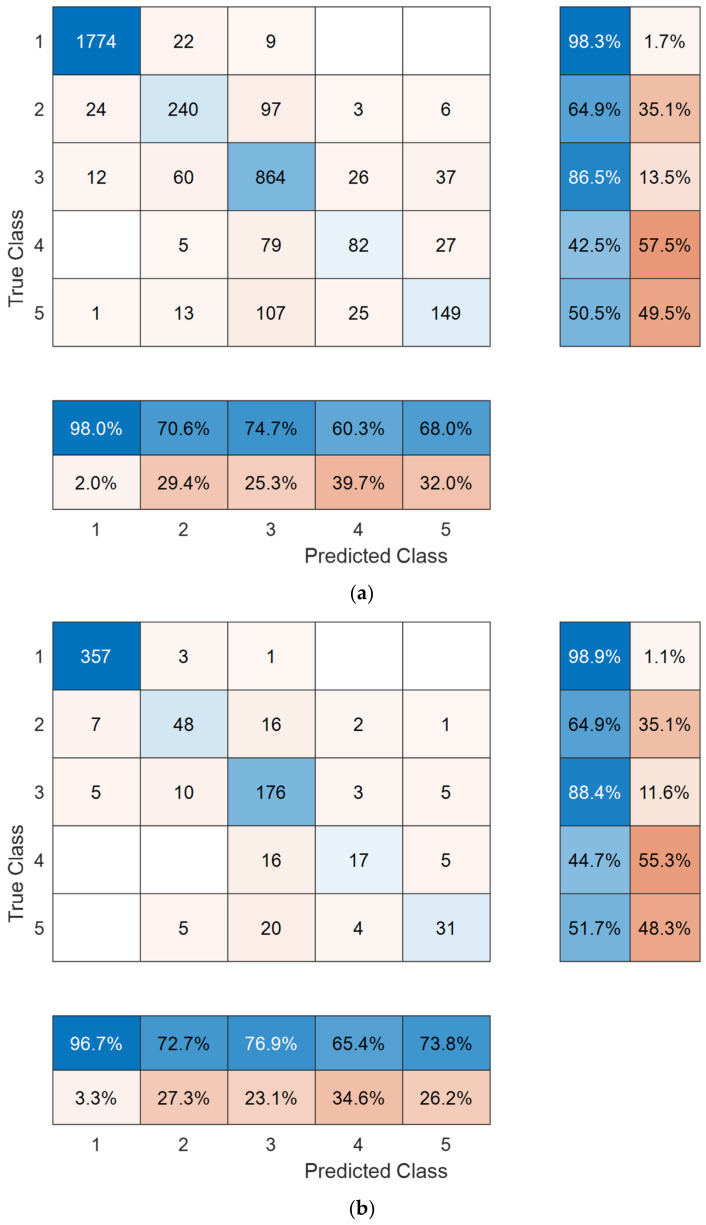
Confusion matrixes for our new dataset obtained using 10-fold cross-validation (**a**) and 80:20 hold-out validation (**b**). Class names: 1, normal; 2, mild diabetic retinopathy; 3, moderate diabetic retinopathy; 4, severe diabetic retinopathy; 5, proliferative diabetic retinopathy.

We further tested our model on the existing five classes of the APTOS 2019 dataset. Congruent with our new DR dataset and Case 1, the best class-wise accuracy was observed in the normal class with either validation strategy (Figure 7). Overall five-class classification performance was good, being numerically superior with hold-out validation across all performance metrics compared with 10-fold CV, e.g., 87.6% versus 84.9% classification accuracy (Table 4). The slightly lower performance compared with Case 1 and our new dataset is not unexpected given the higher number of classes and the imbalanced dataset (as is evident in Figure 7).

### 3.3. Computational Complexity

In order to calculate the computational complexity of our proposal, a big O notation has been used. Our model is a patch-based deep-feature generation model. We have used nonfixed-size patches. Thus, the time complexity of our presented feature generation phase is equal to $O\left(t\times l\times m\times d\right)$. Herein, t,l, and m define the width of the patch, the height of the patch, and the number of patches, and d represents the time complexity coefficient of the pretrained CNN. In the feature selection phase, NCA feature selection function was used, and we defined the time complexity of it as $O\left(n\right),$ and n defines the time complexity coefficient of the NCA function. In the last phase, the SVM classifier has been used, and the time complexity of the SVM is $O\left(c\right)$, where c is the time complexity coefficient of the SVM. The total time burden of our proposal using big O notation is $O\left(t\times l\times m\times d+n+c\right)$. This result demonstrated that our proposal has linear time complexity. Thus, it is a lightweight model.

## 4. Discussion

**Figure 8:**
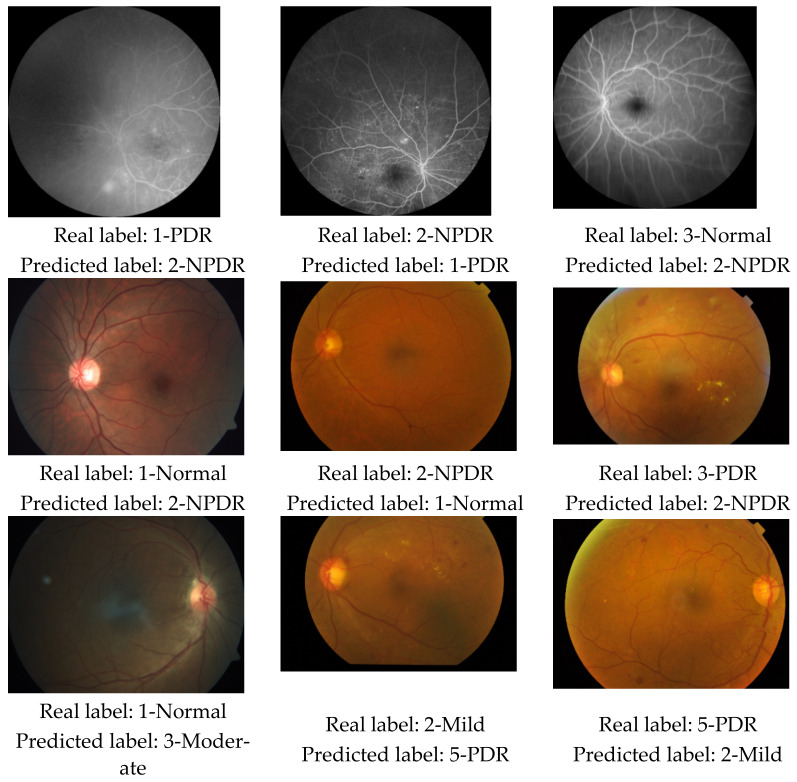
Misclassified samples from our new DR dataset, Case 1 and Case 2 in the top, middle, and bottom rows, respectively.

This study proposed a new DR classification model using patch-based deep-feature extraction. The developed model was tested on the DR images we collected and the established APTOS 2019 dataset. Our new dataset contains three classes (PDR, NPDR, and normal), whereas the APTOS 2019 DR dataset contains five classes (normal, mild DR, moderate DR, severe DR, and PDR). Accordingly, two test scenarios, Case 1 and Case 2, were constructed from the APTOS 2019 dataset. Case 1 contains three classes (normal, NPDR, and PDR), with the NPDR class being constituted by merging the mild DR, moderate DR, and severe DR classes of the APTOS 2019 dataset. In Case 2, the existing classes of the APTOS 2019 dataset were used. The model attained 94.06% and 91.55% three-class accuracy rates for 80:20 hold-out validation and 10-fold CV, respectively, on our new dataset. These results were replicated on the Case 1 dataset, where 93.85% and 92.6% three-class accuracy rates were observed for 80:20 hold-out validation and 10-fold CV, respectively. Finally, the proposed method delivered lower but good five-class classification performance on the Case 2 dataset, where 85.93% and 84.9% for 80:20 hold-out validation and 10-fold CV, respectively, were attained despite the imbalanced dataset. The salutary results validated on different datasets—of note, our new dataset and Case 1 dataset were qualitatively different with predominant PDR (41.1%) and normal (49.3%) images, respectively—underline the feasibility and robustness of the proposed model. Class-wise accuracy was highest with the normal class across all the datasets (97% or more), which highlights the utility of the model as a triage tool to filter out normal DR images. Not unexpectedly, misclassification rates were higher with the five-class classification in Case 2 compared with the three-class classification in Case 1 and our new dataset. Some examples of misclassified DR images are shown in Figure 8.

As can be seen from misclassified fundus image samples (see Figure 8), the symptoms of the disorders are not clear and the quality of these images are low. Thus, our proposal cannot classify these images accurately. Quality image and visibility of the symptoms have affected classification performance of our proposal.

**Figure 9:**
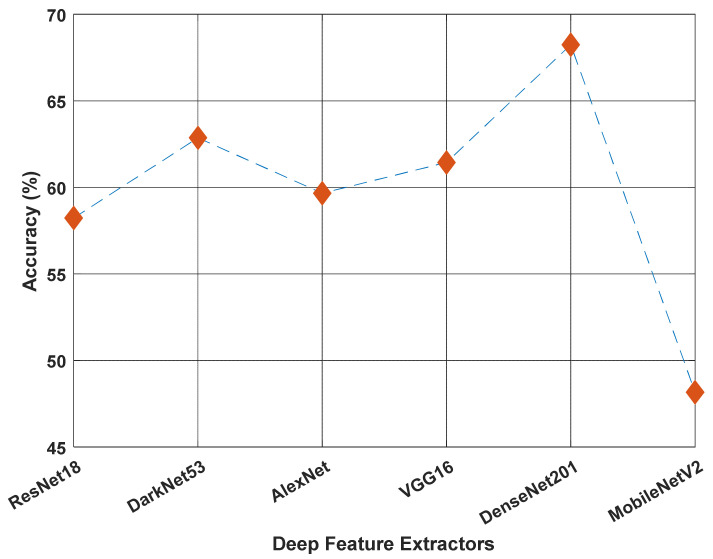
Comparison of the performance of six pretrained deep feature extractors on the ImageNet1k dataset [38]. Neighborhood component analysis was used for each run to select the 1000 most discriminative features, which were then fed to the support vector machine for classification. DenseNet201 yielded the best classification accuracy and was chosen as the deep-feature extractor in our proposed model.

The proposed method used the pretrained DenseNet201 architecture for feature extraction. Features were obtained via the transfer learning method on this architecture. During model development, we tested the classification performance of various pretrained deep learning networks and found DenseNet201 to yield the highest performance (Figure 9). These CNNs were trained on the ImageNet1k dataset [38]. While testing the architectures given in Figure 9, NCA was used as a feature selector, and SVM was used as a classifier. In addition, the 1000 most significant features were selected during the testing process. As a result, the best classification accuracy was obtained using DenseNet201. For this reason, DenseNet201 architecture was used as the deep-feature extractor in our model.

**Figure 10:**
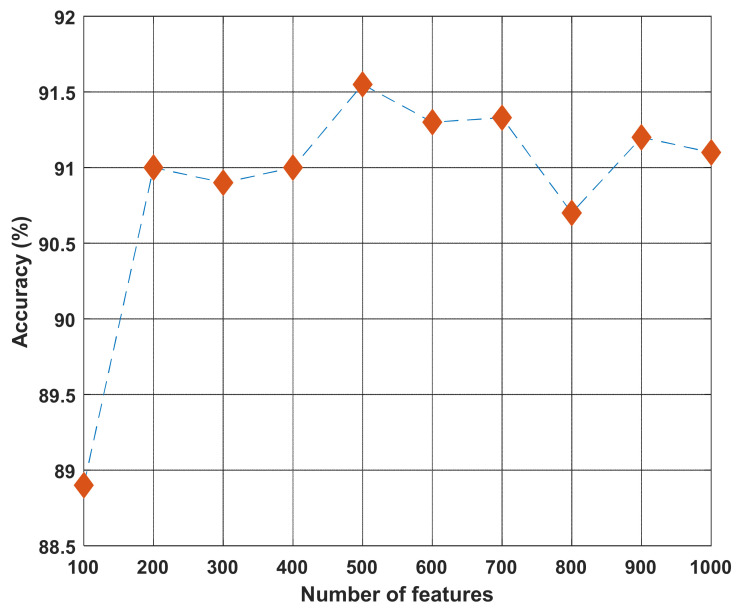
Comparison of classification accuracy by the number of top features selected by neighborhood component analysis (NCA) on the new diabetic retinopathy dataset. For each run, a support vector machine was used for classification. The top 500 NCA-selected features yielded the best classification accuracy, and the number was chosen for our proposed model.

NCA was the feature selection function used in our proposed method. The optimum number of features, 500, had been chosen by the trial-and-error method. During the model development phase, variable numbers of top [100, 1000] features were tested, which showed 500 as the optimum number yielding the highest classification accuracy (Figure 10).

**Figure 11:**
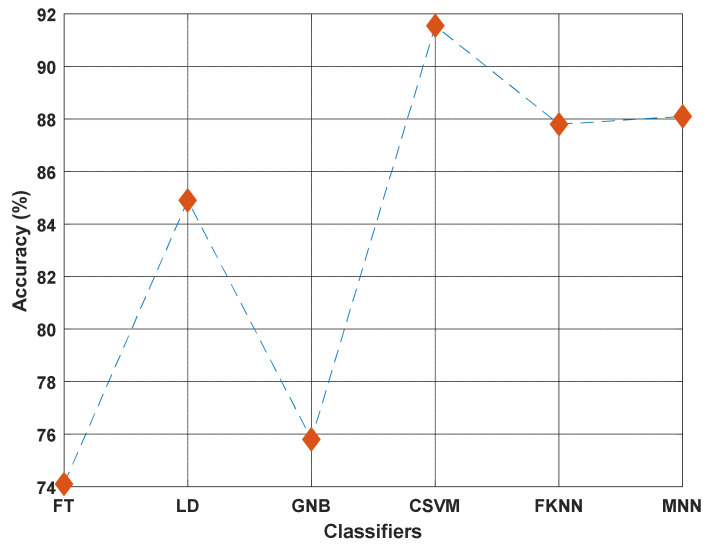
Classification accuracies calculated extractors on our new diabetic retinopathy dataset using six classifiers: fine tree (FT), linear discriminant (LD), Gaussian naïve Bayes (GNB), cubic support vector machine (CSVM), fine k-nearest neighbor (FKNN), and medium neural network (MNN). Neighborhood component analysis was used for each run to select the 500 most discriminative features, which were then fed to respective classifiers for classification. Cubic SVM yielded the best classification accuracy and was chosen as the classifier in our proposed model.

Cubic SVM was the classifier deployed in our proposed model. During model development, various standard classifiers in MATLAB 2021b Classification Learner Toolbox were tested, showing cubic SVM outperforming the rest of the test dataset (Figure 11).

**Figure 12:**
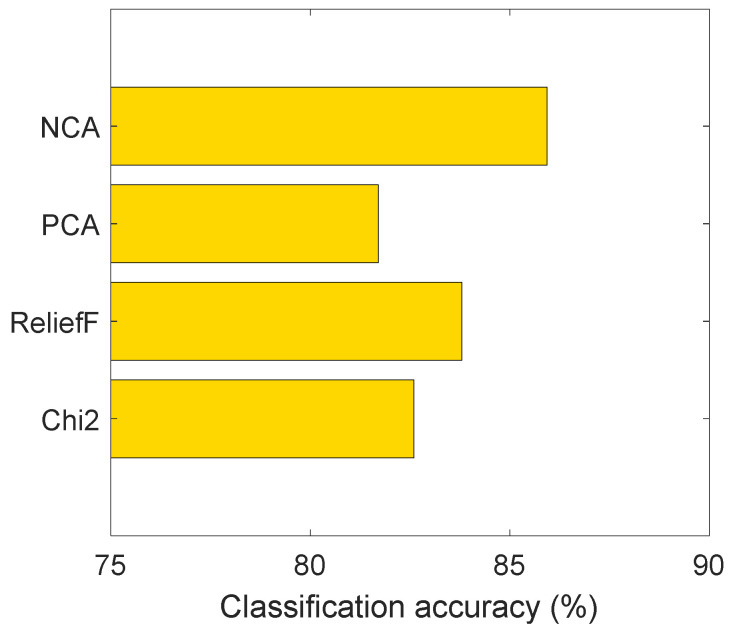
Classification accuracies using Chi2, ReliefF, PCA, and NCA feature selectors on the APTOS dataset.

To choose the most suitable feature selector, we tested four feature selection models, and these feature selection functions are Chi2, ReliefF, PCA, and NCA feature selectors. The calculated results using APTOS dataset with the SVM classifier are demonstrated in Figure 12.

By applying the used four feature selectors, we selected the top 500 features and we classified these features by deploying SVM. Figure 12 depicts that the best accurate feature selector is NCA.

Our proposed model with the chosen components was tested on different datasets, which all produced excellent results. Of note, the salutary results for three-class classification on our new dataset were reproduced on the Case 1 dataset derived from the APROS 2019 dataset, which shares similar DR classes (Table 5). The validation of our model on two different datasets underlines its robustness, which supports its adoption in real-world clinical applications.

We also tested our model for five-class classification on the Case 2 dataset derived from the APTOS 2019 dataset. This is relevant, as many studies in the literature are based on the five-class APTOS 2019 data structure. Comparison with these studies shows that our model has outperformed prior published models, with overall five-class classification accuracy rates of 87.6% and 84.9% on 80:20 hold-out validation and 10-fold CV, respectively (Table 6). In [39], the model attained 79% accuracy using a 10-fold cross-validation method, but the results were obtained with data augmentation. In [40], the model attained 81% accuracy with data augmentation and end-to-end learning. Of note, end-to-end training of convolutional neural networks is computationally demanding and time-consuming, which is an important factor to consider when implementing the model. Podapati et al. [41] employed similar verification techniques as our model and achieved a slightly lower 80.96% accuracy with 80:20 hold-out validation. In [42], the CNN-based model attained 83.09% accuracy using only 10% of the data for testing. In [43], a proposed model based on Inception and ResNet, which employed data augmentation and time-consuming end-to-end training, attained 82.18% accuracy.

As can be seen from Table 6, most of the models (see Majumder and Kehtarnavaz [40], Kassani et al. [42], Taufiqurrahman et al. [39], and Gangwar and Ravi [43]) applied data augmentation to overcome image classification on the unbalanced dataset. However, data augmentation is not a good way to show the performance of the model. Moreover, these models were used for end-to-end deep learning, and the time complexity of the end-to-end deep learning is exponential in Gangwar and Ravi [43]’s method. Therefore, they propose a hybrid CNN to obtain classification results. They used data augmentation, and their presented deep learning model is an end-to-end. Their model has exponential computational complexity, their results are augmented results, and they used hold-out validation to obtain results, since they presented nonrobust results. They attained 82.18% classification accuracy, but our model reached 84.90% with 10-fold CV without end-to-end deep learning (we only used pretrained DenseNet201) and data augmentation. Moreover, Kassani et al. [42] presented an Xception-Net-based model (their model is the best of others) and they reached 83.09% classification accuracy by deploying hold-out validation 70:20:10. We deployed this validation model and our proposal reached 85.52% testing classification accuracy. Our model extracted features efficiently with the transfer learning approach. Discriminative features were selected with NCA, and selected features were classified with shallow classifier cubic SVM. Of note, neither data augmentation/reduction nor complicated preprocessing techniques were utilized.

The highlights of our work are listed below:

- A new DR image dataset collected comprised of three classes: normal, NPDR, and PDR.
- A new patch-based deep-feature extraction method was proposed that used pretrained DenseNet201 architecture to generate many deep features.
- The optimal number of top features was selected by applying NCA, which were fed to a shallow cubic SVM classifier.

## 5. Conclusions

In this paper, a new model using a novel patch-based deep-feature generator demonstrated high classification accuracy for DR. The proposed method, inspired by vision transformer (ViT) and multilevel perceptron-mixer (MLP-mixer) methods, divides the image into horizontal and vertical patches. ViT and MLP-mixer uses fixed-sized square patches for feature extraction. In this research, we have used rectangle patches that are not fixed-size, since we used vertical and horizontal versions of these patches to generate hidden deep patterns. The raw image and the created patches generate features by deploying pretrained DenseNet201. Using this strategy, global and local high-leveled features were generated from a fundus image. Then, the top discriminative features were selected using NCA, and input to shallow cubic SVM for classification using 10-fold CV and 80:20 hold-out validation strategies. The model attained excellent accuracy results of more than 90% for three-class (normal, NPDR, and PDR) classification on both our new dataset and Case 1 dataset derived from the APTOS 2019 dataset. This underscores the reproducibility and robustness of the model. For the five-class classification on Case 2 of the APTOS 2019 dataset, the model attained slightly lower but still excellent accuracy rates that outperformed other published studies of DR classification using the same dataset. Of note, unlike many of these prior studies, our model is simple and employed neither data augmentation/reduction (despite the imbalanced Case 2 dataset) nor complicated preprocessing techniques.

The accuracy and efficiency of our model are important advantages that support its implementation in the clinic to assist doctors in the screening of DR, as well as the grading of its severity among diabetic patients. Moreover, our proposed patch division model can be used with transformers to propose new-generation computer vision models. Our proposal is a parametric image classification model, since we used eight rectangular patches, DenseNet201, NCA, and SVM methods together. In the near future, other types of patches, feature extraction, feature selection, and classification methods can be used for this architecture, and new-generation image classification methods can be proposed. Moreover, our proposal can test on the bigger datasets.

## Figures and Tables

**Table 1:** Summary of a nonsystematic review of recent studies on machine-learning-enabled automated classification of diabetic retinopathy using fundal photography.

Study	Method	Dataset	Classes (Validation)	Results (%)
Arunkumar and Karthigaikumar 2017 [15]	DBN-based feature extraction, SVM	ARIA [16]	Normal; DR, age-related macular degeneration	Acc 96.73 Sen 79.32 Spe 97.89
Abbas et al., 2017 [17]	GLOH, principal component analysis, deep neural network	DIARETDB1 [18]	Normal, mild NPDR, moderate NPDR, severe NPDR, PDR (10-fold CV)	AUC 92.4 Sen 92.18 Spe 94.5
Krause et al., 2018 [19]	Custom CNN	Own dataset, Messidor-2 [20,21]	Not available	AUC 98.6
Chetoui et al., 2018 [22]	LTP, LESH, and SVM	Messidor [20,21]	DR, non-DR (10-fold CV)	Acc 90.04 AUC 93.1
Orlando et al., 2018 [23]	CNN and handcrafted feature extraction, random forest	Messidor [20,21]	Lesion detection	AUC 93.47 Sen 97.21
Zeng et al., 2019 [24]	InceptionV3-based CNN	Kaggle [25]	DR, non-DR (80:20 hold-out)	AUC 95.1
Ali et al., 2020 [26]	Texture analysis (histogram, wavelet, co-occurrence, run-length matrix), logistic model tree	Own dataset	Normal, mild NPDR, moderate NPDR, severe NPDR, PDR (10-fold CV)	Acc 99.73 Cohen’s kappa 99.67
Gayathri et al., 2021 [27]	Multipath CNN, ResNet-50, and VGG-16-based feature extraction, classifiers (SVM, random forest, J48)	3 datasets: IDRiD [28], Kaggle [25], Messidor [20,21]	IDRiD: Normal, mild NPDR, moderate NPDR, severe NPDR, PDR; Kaggle: Normal, mild NPDR, moderate NPDR, severe NPDR, PDR Messidor: Normal, mild DR, moderate DR, severe DR (10-fold CV)	Overall Acc 99.62 Cohen’s kappa 99.5
Mahmoud et al., 2021 [29]	HIMLA (preprocessing, segmentation, feature extraction, and classification)	Chase_DB1 [30]	DR, non-DR	Acc 96.62 Sen 95.31 Spe 96.88
Math and Fatima 2021 [31]	Custom CNN	Kaggle [25], DIARETDB1 [18]	Normal, mild, moderate, NPDR, PDR	AUC 96.3 Sen 96.37 Spe 96.37

Definitions in the Table 1: Acc, accuracy; AUC, area-under-curve; CNN, convolutional neural network; CV, cross-validation; DBN, deep belief neural network; DR, diabetic retinopathy; GLOH, gradient location orientation histogram; HIMLA, hybrid inductive machine learning algorithm; LESH, local energy-based shape histogram; LTP, local ternary pattern; NPDR, non-proliferative diabetic retinopathy; PDR, proliferative diabetic retinopathy; Sen, sensitivity; Spe, specificity; SVM, support vector machine.

**Table 2:** Overall three-class classification performance on our new diabetic retinopathy dataset.

	Results (%)	
Performance Metric	10-Fold CV	80:20 Hold-Out Validation
Accuracy	91.55	94.06
Unweighted average recall	92.67	94.45
Unweighted average precision	92.77	94.74
Average F1	92.70	94.59
Cohen’s kappa	86.34	90.38
Geometric mean	92.61	94.45

**Table 3:** Overall three-class classification performance on Case 1 of the APTOS dataset.

	Results (%)	
Performance Metric	10-Fold CV	80:20 Hold-Out Validation
Accuracy	92.60	93.85
Unweighted average recall	79.64	80.60
Unweighted average precision	86.63	90.90
Average F1	82.06	83.78
Cohen’s kappa	86.74	88.94
Geometric mean	75.28	76.04

**Table 4:** Overall five-class classification performance on Case 2 of the APTOS dataset.

	Results (%)	
Performance Metric	10-Fold CV	80:20 Hold-Out Validation
Accuracy	84.90	85.93
Unweighted average recall	68.53	69.72
Unweighted average precision	74.32	77.11
Average F1	70.75	72.51
Cohen’s kappa	76.91	78.37
Geometric mean	65.25	66.61

**Table 5:** Comparison of model performance model on our new DR dataset and Case 1 dataset.

	Automated Diabetic Retinopathy Detection Model			
	Our New DR Dataset		Case 1 Created Using APTOS 2019	
Dataset Information	3 Class: PDR/NPDR/Normal 2355 Images		3 Class: Normal/NPDR/PDR 3662 Images	
Performance Metric	10-Fold CV	80:20 Hold-Out	10-Fold CV	80:20 Hold-Out
Accuracy	91.55	94.06	92.60	93.85
Unweighted average recall	92.67	94.45	79.64	80.60
Unweighted average precision	92.77	94.74	86.63	90.90
Average F1	92.70	94.59	82.06	83.78
Cohen’s kappa	86.34	90.38	86.74	88.94
Geometric mean	92.61	94.45	75.28	76.04

**Table 6:** Comparison of model performance on Case 2 dataset with prior studies using the APTOS 2019 dataset.

Author(s)	Method	Key Points	Results (%)
Majumder and Kehtarnavaz 2021 [40]	Modified DenseNet-based squeeze excitation densely connected multitasking network (MSEDenseNet)	90:10 hold-out validation Data augmentation End-to-end learning	Acc 81.0 Pre 67.0 Rec 59.0 F1 61.0 Kap 84.0
Podapati et al., 2020 [41]	Feature extraction using VGG16′s fc1 and fc2 layers and XCeption’s global average pooling layers, deep neural network	80:20 hold-out validation Training using combined features	Acc 80.96 Kap 71.1
Kassani et al., 2019 [42]	Xception CNN architecture	70:20:10 hold-out validation Min-pooling and normalization-based image pre-processing	Acc 83.09 Sen 88.24 Spe 87.0
Taufiqurrahman et al., 2020 [39]	MobileNetV2 CNN architecture, SVM	10-fold cross validation Data augmentation	Acc 79.0 Kap 88.0
Gangwar and Ravi 2021 [43]	Inception and ResNet-based custom CNN	75:25 hold-out validation Data augmentation End-to-end learning	Acc 82.18
Our model, Case 2	Feature extraction with DenseNet, feature selection with neighborhood component analysis, cubic SVM classifier	10-fold cross validation 80:20 hold-out validation No data augmentation Validation with three datasets (our new dataset, Case 1, and Case 2 derived from APTOS 2019 dataset)	10-fold CV
			Acc 84.90 UAR 68.53 UAP 74.32 F1 70.75 Kap 76.91 GM 65.25
			80:20 hold-out
			Acc 85.93 UAR 69.72 UAP 77.11 F1 72.51 Kap 78.37 GM 66.61

Acc, accuracy; CNN, convolutional neural network; F1, F1-score; GM, geometric mean; Kap, Cohen’s kappa; Pre, precision; Rec, recall; Sen, sensitivity; Spe, specificity; SVM, support vector machine; UAP, unweighted average precision; UAR, unweighted average recall.

## Data Availability

The data presented in this study are available on request from the corresponding author. The data are not publicly available due to restrictions regarding the Ethical Committee Institution.
